# The Effect of the Modification of Perioperative Anti-Rheumatic Medications on Early Complications After Total Ankle Arthroplasty for Severe Rheumatoid Arthritis

**DOI:** 10.3390/jcm14207280

**Published:** 2025-10-15

**Authors:** Jin Woong Yi, Sung-Hoo Kim, Seung-Min Bang, Byung-Ki Cho

**Affiliations:** 1 Department of Orthopaedic Surgery, College of Medicine, Konyang University, Daejeon 35365, Republic of Korea; woong@kyuh.ac.kr; 2 Department of Orthopaedic Surgery, Chungbuk National University Hospital, Cheongju 28644, Republic of Korea; hoo414414@hanmail.net (S.-H.K.); ddunmin2@naver.com (S.-M.B.); 3 Department of Orthopaedic Surgery, College of Medicine, Chungbuk National University, Cheongju 28644, Republic of Korea

**Keywords:** ankle, rheumatoid arthritis, arthroplasty, complication, anti-rheumatic medication

## Abstract

**Background/Objectives**: The potential risk of postoperative complications related to chronic inflammation and immunosuppressive therapy is still an ongoing concern for patients with rheumatoid arthritis (RA). This retrospective comparative study reports the outcomes, including early complications after total ankle arthroplasty (TAA), for end-stage RA, and the effects of the modification of perioperative anti-rheumatic medications. **Methods**: A total of 18 patients (the controlled group) with and 16 (the uncontrolled group) without the modification of perioperative anti-rheumatic medications (according to an established guideline used in total hip and knee arthroplasty) were followed for 2 years after three component mobile-bearing TAA. Clinical evaluations consisted of the American Orthopaedic Foot and Ankle Society (AOFAS) scores, the Foot and Ankle Outcome Score (FAOS), and the ASEPSIS score for wound assessment. Additionally, clinical and radiological complications, and reoperation rates were investigated. **Results**: In the postoperative follow-up evaluation at 2 years, there were no significant differences in the AOFAS and FAOS scores between the two groups. In postoperative wound monitoring, using the ASEPSIS scoring system, the controlled group demonstrated the better wound healing up to 4 weeks postoperatively, compared to the uncontrolled group (3.1 vs. 6.8 points, *p* = 0.003). In terms of early postoperative complications, there was a significant difference in delayed wound healing rates between the two groups (0 vs. 25%, *p* = 0.039). Up to 2 years postoperatively, there were no significant differences in the radiological complications and reoperation rates between the two groups. **Conclusions**: In TAA for end-stage RA, the modification of perioperative anti-rheumatic medications based on the specified guideline appears to be one of the effective solutions to reduce early postoperative wound complication.

## 1. Introduction

With a rapidly aging population, there is growing interest in the prevention and treatment of ankle arthritis. Among the surgical options for end-stage ankle arthritis, the most commonly performed procedures are total ankle arthroplasty (TAA) and ankle arthrodesis. Ankle arthrodesis has long been considered the standard treatment for end-stage arthritis, partly due to the high failure rates associated with early TAA prostheses [1,2]. However, recent advances in surgical techniques and implant designs have led to improved clinical outcomes [3,4]. Compared to arthrodesis, TAA offers the advantages of preserving joint motion, allowing for more normal gait, better adaptability on uneven surfaces, and reduced biomechanical stress on adjacent joints, potentially preventing the development of secondary arthritis [5,6,7].

As with other orthopedic conditions, appropriate patient selection is critical to achieving satisfactory outcomes and minimizing complications following TAA. In fact, patient selection may have an even greater influence on outcomes in TAA than in other joints. Commonly accepted indications for TAA include end-stage arthritis in middle-aged or elderly patients with minimal deformity in the ankle and hindfoot, low demands of physical activity, preserved joint range of motion, intact neurovascular function, good bone quality, and non-obese body habitus. Primary osteoarthritis is generally considered to be a more favorable indication than post-traumatic arthritis [8]. Conversely, severe deformities of the ankle or hindfoot, peripheral vascular disease, Charcot neuroarthropathy, recent infection, poor soft tissue condition, advanced avascular necrosis of the talus, and joint hypermobility are considered relative contraindications [2,8,9].

Patients with rheumatoid arthritis (RA) often present with poor soft tissue and bone quality due to the chronic inflammatory nature of the disease and the long-term use of immunosuppressive disease-modifying anti-rheumatic drugs (DMARDs), including corticosteroids. These factors are known to increase the risk of postoperative complications [10,11,12,13]. Recent studies evaluating the impact of DMARDs on complication rates in foot and ankle surgery have found no significant correlation between perioperative DMARD use and complication rates [14]. However, prolonged surgical duration and preoperative peripheral neuropathy have been shown to significantly increase the risk of complications. TAA in RA patients, compared to other foot surgeries, generally involves longer operation times and larger incisions, and the resulting complications can greatly influence overall outcomes [15,16]. Therefore, caution is needed when applying these study results.

While evidence-based guidelines exist for perioperative DMARD management in total hip or knee arthroplasty [17], there are currently no specific guidelines for TAA. In most cases, perioperative medication management is performed in collaboration with rheumatology specialists based on the patient’s condition. Although long-term corticosteroid use may be considered a relative contraindication to TAA, there is limited and conflicting evidence on whether end-stage RA itself serves as a prognostic factor for TAA outcomes, or whether differences exist in clinical outcomes compared to patients with degenerative arthritis.

This retrospective comparative study aimed to evaluate the outcomes, including early complications, after TAA for end-stage RA, and the effects of the modification of perioperative anti-rheumatic medications on the incidence of postoperative complications.

## 2. Materials and Methods

### 2.1. Study Design

Prior to the subjects’ enrollment, a power analysis was performed to determine the appropriate sample size in order to obtain statistically significant results. We performed a verification study (with 90% power) to assess the hypothesis that TAA with the modification of perioperative anti-rheumatic medications would show a favorable effect on the postoperative complications than those without modification. The 95% confidence interval (a type I error rate of 0.05) was used to evaluate whether the difference in the ASEPSIS scoring system was within a margin of noninferiority. The margin of noninferiority was 7 points (a delta of 7 points in the ASEPSIS score) and the estimated dropout rate was 5%. The margin of noninferiority and the dropout rate were determined on the basis of a pilot study. Eventually, the sample size required to make statistically significant results was 15 subjects in each group.

### 2.2. Study Subjects

**Figure 1:**
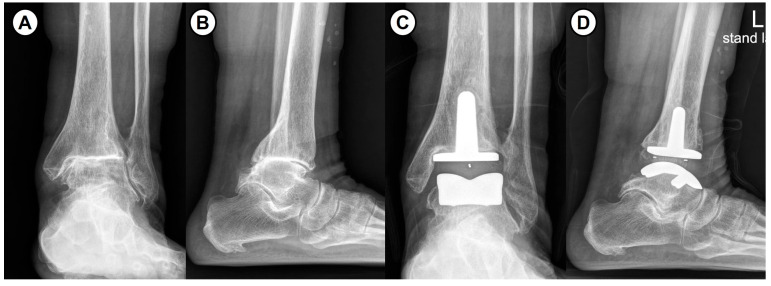
(**A**,**B**) Preoperative and (**C**,**D**) postoperative radiographs in a 68-year-old female with total ankle arthroplasty for end-stage rheumatoid arthritis.

**Figure 2:**
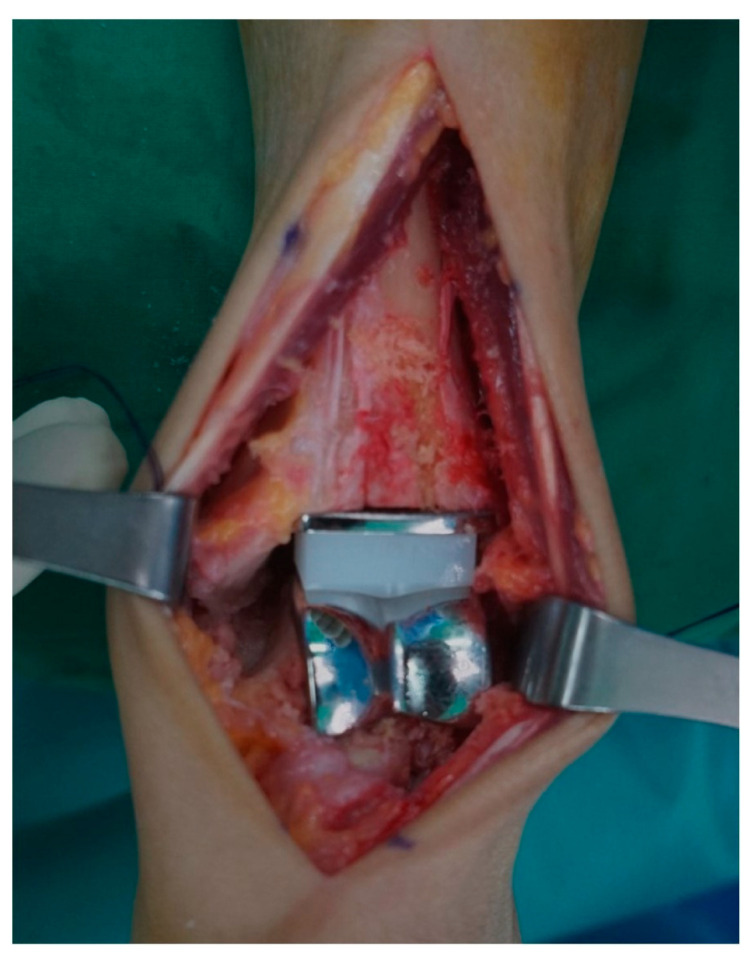
Intraoperative photograph showing the three-component mobile-bearing total ankle arthroplasty (Zenith™).

From January 2017 to June 2023, a total of 34 patients diagnosed with end-stage ankle RA who underwent TAA and were followed for 2 years were included in this study. A total of 16 patients without the modification of perioperative anti-rheumatic medications from January 2017 to April 2020 were classified into the uncontrolled group. Consecutively, 18 patients with the modification of perioperative anti-rheumatic medications from May 2020 to June 2023 were classified into the controlled group. All surgeries were performed by a single surgeon using a third-generation three-component mobile-bearing implant system (Zenith™; Corin, Cirencester, UK) (Figure 1 and Figure 2). The modification of perioperative anti-rheumatic medications was performed according to an established guideline [17] used in total hip and knee arthroplasty.

All patients had a history of long-term disease-modifying anti-rheumatic drug (DMARD) use (mean duration, 9.6 years) and presented with severe ankle pain and gait disturbance lasting more than 6 months despite conservative treatment. Patients with peripheral vascular disease, advanced avascular necrosis of the talus, recent active infection, Charcot neuroarthropathy, or neuromotor/sensory dysfunction were excluded from TAA. Furthermore, patients requiring additional corrective supramalleolar osteotomy due to varus or valgus deformities with coronal malalignment exceeding 25 degrees were excluded from this study. This study was approved by the ethics committee of the Institutional Review Board of our institution (CBNUH 002-07-2024) and informed consent was given by all participants.

At the time of surgery, the mean age of the patients was 63.2 years (range, 54 to 73 years), and the mean duration of ankle pain was 65.1 months (range, 11 to 118 months). This study included 31 female and 3 male patients. The mean body mass index (BMI) was 25.1 kg/m^2^, and 8 patients had T-scores below −2.5 on bone mineral density tests measured at the spine or hip. All patients exhibited varus deformities associated with end-stage rheumatoid ankle arthritis, with mild to moderate deformities less than 20 degrees in all but 3 patients. No significant radiographic signs of progressive arthritis were noted in the ipsilateral hindfoot or midfoot joints. However, 2 patients had contralateral planovalgus deformity due to severe rheumatoid arthritis involving the subtalar and transverse tarsal joints. Preoperative computed tomography (CT) was performed for all patients, and 5 patients showed considerable subchondral cysts or erosive bone defects that required additional bone grafting during surgery.

Regarding comorbidities, 9 patients had hypertension, and 5 had diabetes mellitus, all of whom were medically well-controlled with regular medication. The medications for RA included nonsteroidal anti-inflammatory drugs (NSAIDs) for 31 patients, conventional DMARDs for 25 patients, low-dose glucocorticoids (≤20 mg/day) for 13 patients, high-dose glucocorticoids for 5 patients, and biologic agents for 14 patients.

### 2.3. Modification of Perioperative Anti-Rheumatic Medications

Medication adjustments for all patients in the controlled group were based on the clinical guidelines proposed by Goodman et al. [17] for the perioperative management of anti-rheumatic drugs in hip and knee arthroplasty. NSAIDs and DMARDs, including methotrexate, sulfasalazine, hydroxychloroquine, and leflunomide, were continued without interruption throughout the perioperative period. Different glucocorticoid molecules (prednisone, methylprednisolone, and dexamethasone) require distinct dosing and tapering regimens. Prednisone, which is the most frequently used oral glucocorticoid, was gradually tapered starting from the time surgical planning was confirmed, and surgery was performed only when the dosage had been adjusted to 20 mg/day or less. This dosage was maintained postoperatively until complete wound healing was confirmed. Biologic agents, including golimumab, adalimumab, etanercept, abatacept, infliximab, and certolizumab, were withheld at the end of their dosing cycle prior to surgery. These agents were resumed at least two weeks after surgery, only if satisfactory wound healing was observed and there were no signs of infection.

All patients in the uncontrolled group maintained their anti-rheumatic medications without any specific modification by physicians.

### 2.4. Clinical and Radiological Evaluation Methods

Clinical outcomes were assessed using the American Orthopaedic Foot and Ankle Society (AOFAS) Ankle–Hindfoot Score, and the Foot and Ankle Outcome Score (FAOS). The AOFAS score was evaluated by the principal investigator, while the FAOS, which is a patient-reported outcome measure, was self-administered. A co-investigator provided further explanation only when patients had difficulty understanding specific questions. The FAOS [18] consists of five subscales: pain (9 items), symptoms (7 items), activities of daily living (17 items), sports and recreational function (5 items), and quality of life (4 items). Each question is rated on a 5-point Likert scale: extreme (1 point), severe (2 points), moderate (3 points), mild (4 points), and normal (5 points). The total scores were converted to a 100-point scale for analysis. All clinical evaluations were conducted within one month preoperatively, every six months during the first postoperative year.

Radiographic evaluations were performed using serial weight-bearing anteroposterior and lateral plain radiographs before and after surgery. These assessments evaluated the deformities of the tibiotalar joint, the alignment of the implant components, the progression of degenerative arthritis in adjacent joints (subtalar, transverse tarsal, and Lisfranc joints), implant loosening or subsidence, and the presence of heterotopic ossification. Malalignment of the implant was defined as an angle greater than 10° between the articular surface of the tibial component and the anatomical axis of the tibia. Loosening or subsidence was defined based on the criteria described by Hintermann et al. [19] for the tibial component, a change in alignment exceeding 2° or migration over 2 mm; and for the talar component, a change in alignment exceeding 5° or migration over 5 mm. For patients showing a radiolucent line of 2 mm or more around the metal implant, follow-up radiographs were taken at 2-month intervals. If progressive radiolucency was observed, computed tomography (CT) was performed to measure the extent of osteolysis and to assess the need for revision procedures, such as debridement with bone grafting or polyethylene liner exchange. All radiological evaluations were independently performed by two researchers using the digital PACS imaging system.

### 2.5. Postoperative Wound Assessment

Postoperative wound healing was assessed using the ASEPSIS scoring system [20]. All wound evaluations were performed by the principal investigator every week up to 4 weeks postoperatively. The ASEPSIS scoring system consists of 4 items evaluating wound condition (serous exudate, erythema, purulent exudate, and separation of deep tissues) and 6 items related to wound complications (prescription of antibiotics, drainage under local anesthesia, debridement under general anesthesia, bacterial isolation, hospital stay prolonged >14 days, and development of pus). A lower score indicates a better wound healing status.

### 2.6. Statistical Analysis

The statistical analysis was performed using SPSS program (version 21.0; SPSS Inc., Chicago, IL, USA), and a *p*-value of less than 0.05 with a confidence interval of 95% was considered to indicate statistical significance. All data showed a normal distribution using the Kolmogorov–Smirnov test. A comparison of the preoperative and postoperative outcomes in the same patients was conducted using the Wilcoxon signed-rank test. The outcomes between the controlled and the uncontrolled groups were compared using the Mann–Whitney test. Categorical data, such as patient demographics and complication rates, were compared using the Fisher exact test.

## 3. Results

### 3.1. Comparison of Demographic Characteristics and Operative Procedures

The patients’ baseline demographic information is listed in Table 1. There were no significant differences in demographic and clinical characteristics between the two groups. Data regarding the operative procedures are listed in Table 2. There were no significant differences in tourniquet time and additional procedures combined with TAA between the two groups.

### 3.2. Comparison of Clinical Outcomes

In both groups, the mean AOFAS and FAOS scores improved significantly compared with the preoperative measurement (*p* < 0.001) (Table 3). In the postoperative evaluation at 2 years, there were no statistically significant differences in the AOFAS and FAOS scores between the two groups. The mean sagittal range of motion (ROM) of the ankle increased slightly from 39.6° preoperatively to 42.5° postoperatively in the controlled group, and from 39.2° to 42.3° in the uncontrolled group. There was no statistically significant difference in the changes of ankle ROM between the two groups (*p* = 0.887). At 2 years postoperatively, 16 patients (88.9%) in the controlled group and 14 patients (87.5%) in the uncontrolled group reported significant pain relief during gait, with a visual analogue scale (VAS) score of 2 or less (*p* = 0.758).

### 3.3. Comparison of Postoperative Complications

Postoperative complications included marginal skin necrosis in three patients, delayed wound healing (>4 weeks) in four patients, medial malleolar fracture in two patients, superficial wound infection in one patient, deep peroneal nerve symptoms in one patient, malalignment (≥10°) of the tibial component in one patient, subsidence (>5 mm) of the tibial component in two patients, heterotopic ossification in two patients, soft tissue or spur impingement in three patients, and progressive arthritis of the adjacent joint (talonavicular) in one patient. Except for delayed wound healing, there were no statistically significant differences in the postoperative complication rates the between the two groups (Table 4). Delayed wound healing (>4 weeks) was found in only four patients in the uncontrolled group, with a statistically significant difference compared to the controlled group (*p* = 0.039). In the controlled group, no rheumatoid flare-up occurred during the steroid interruption according to the modification of perioperative anti-rheumatic medications.

All marginal skin necrosis, delayed wound healing, and superficial wound infection were treated successfully after continuous wound care (dressing, debridement, and repair) and prophylactic antibiotics, without the need for reoperation. One patient with deep peroneal nerve injury experienced neuralgia and paresthesia in the first web space, which resolved spontaneously within six6 months without specific treatment. Two nondisplaced medial malleolar fractures were incidentally detected on radiographs at 2 weeks postoperatively and achieved successful union after 6 weeks of cast immobilization. Three patients developed medial soft tissue or spur impingement during the follow-up period. They experienced persistent medial ankle pain during gait, with tenderness along the articular surface of the medial malleolus and a snapping during ROM. Arthroscopic gutter debridement and spur trimming were performed at 8–14 months postoperatively, leading to symptom resolution. Although subsidence (>5 mm) of the tibial component in two patients was detected, we continued to monitor based on no progression of further collapse, without additional bone graft or implant revision. Heterotopic ossification was noted in two patients, but none required revision surgery due to associated symptoms. One case with progressive arthritis in the adjacent talonavicular joint was observed, showing moderate radiographic changes without notable clinical symptoms. In terms of reoperation rates, there were no statistically significant differences between the two groups (*p* = 0.365); two cases (11.1%) in the controlled group vs. one case (6.3%) in the uncontrolled group.

### 3.4. Comparison of Postoperative Wound Healing

On periodic wound assessment after surgery, there were statistically significant differences in the ASEPSIS score between the two groups. The ASEPSIS scores were 13.8 points for the controlled group and 17.1 points for the uncontrolled group at 2 weeks postoperatively (*p* = 0.014), 9.2 and 14.7 points at 3 weeks postoperatively (*p* < 0.001), 3.1 and 6.8 points at 4 weeks postoperatively (*p* = 0.003) (Table 5).

## 4. Discussion

The most significant finding of this study is that TAA with the standardized perioperative modification of anti-rheumatic medications for end-stage RA resulted in favorable short-term clinical outcomes with a relatively low rate of early complications. TAA has been reported to yield clinical outcomes and survival rates comparable to the traditional gold-standard treatment, ankle arthrodesis, in patients with end-stage ankle arthritis. With the continued development of prosthetic designs and surgical techniques, the indications for TAA have broadened, and factors once considered relative contraindications are increasingly being overcome.

Patients with RA are generally considered to be at higher risk for postoperative complications due to chronic immunosuppressive treatment, including corticosteroids, which often compromise soft tissue and bone quality [21]. Consequently, RA has traditionally been reported as a relative contraindication for TAA in some reports [10,11,12]. Since wound healing problems and infection-related complications can critically affect the outcomes and survivorship of TAA prostheses, careful patient selection is especially crucial in RA cases. Wixted et al. have reported that patients with RA show poorer patient-reported outcome scores compared to those with the OA and post-traumatic arthritis [22]. Common pharmacologic treatments for RA include NSAIDs, DMARDs, corticosteroids, and biologic agents. While the optimal combination of these therapies can effectively manage most RA patients, the treatment paradigm has shifted from a stepwise “pyramid” approach to more aggressive early intervention [23]. However, immunosuppression associated with these treatments can increase the risk of postoperative infections, delay wound healing, and negatively impact bone formation [15,24,25]. Although various new anti-rheumatic agents have emerged, none are free from adverse effects. Thus, the perioperative adjustment of these medications may be essential in patients undergoing orthopedic procedures.

Striking a balance between the risk of postoperative complications from continued medication use and the risk of disease flare-up from discontinuation remains a challenge, and current evidence remains conflicting [26]. Somayaji et al. [24] analyzed the risk factors for postoperative wound infections in 259 patients who underwent hip or knee arthroplasty and identified high-dose corticosteroid use (≥15 mg/day of prednisone), underweight status, and coronary artery disease as significant risk factors, whereas the type of DMARDs, age, and sex were not associated with infection risk. Grennan et al. [25] found no significant difference in postoperative complication rates between patients who continued or discontinued methotrexate in a cohort of 388 orthopedic surgery patients. Weinreb et al. [27], in a study of 34,970 patients undergoing shoulder arthroplasty, found higher rates of blood transfusion and longer hospital stays in RA patients compared to non-RA patients, but reported no significant differences in the incidence of other complications. By contrast, Newton reported a higher complication rate associated with long-term corticosteroid use and advocated for indicating TAA to only RA patients who were not on long-term steroid therapy [28].

Goodman et al. recently proposed evidence-based guidelines for the perioperative management of anti-rheumatic medications in hip and knee arthroplasty [17]. However, no standard guidelines currently exist for TAA, and perioperative management is typically individualized in consultation with rheumatology specialists or not specifically controlled. In this study, the controlled group with the perioperative modification of anti-rheumatic medications demonstrated only one patient (5.6%) who experienced marginal skin necrosis, and no cases of wound infection or delayed wound healing (>4 weeks). While prior studies on TAA in RA patients lack detailed descriptions of perioperative medication management, the relatively low incidence of wound complications in this study suggests that the proper modification of anti-rheumatic medications may contribute to a lower complication rate.

With a rapidly aging population, the number of patients with end-stage ankle arthritis is increasing. It is estimated that 15–52% of adult-onset RA patients and up to 70% of juvenile-onset RA patients develop ankle arthritis [29]. As the use of TAA in RA patients continues to rise, the need for evidence-based guidelines on the perioperative management of anti-rheumatic medications in TAA is increasingly urgent. Wound-related complications, along with medial malleolar fractures, are among the most common early adverse events following TAA, with a reported incidence ranging from approximately 4% to 14% [19]. Such complications warrant particular caution in patients with end-stage RA who have been undergoing long-term anti-rheumatic medication therapy. Minimizing soft tissue trauma during surgery and reducing operative and tourniquet times are crucial strategies to decrease the risk of wound complications. In cases requiring additional procedures, such as hindfoot realignment osteotomies, a staged surgical approach is often recommended [30].

Postoperative infection rates following TAA range from 2% to 12%, comparable to those seen in hip or knee arthroplasty [31,32]. However, due to the immunosuppressive effects of anti-rheumatic drugs, RA patients are at relatively increased risk. Therefore, wound management and the duration of prophylactic antibiotic use should be tailored to each patient’s individual condition. It has been reported that patients with comorbidities, such as diabetes, obesity, hypothyroidism, or peripheral vascular disease, exhibit higher infection rates [33]. In cases of deep infection, complex revision procedures, such as prosthesis removal with two-stage reimplantation using antibiotic-loaded cement spacers or conversion to arthrodesis, are often required, significantly affecting the prognosis of TAA. Kofoed and Sørensen have reported that RA patients undergoing TAA may experience deep infections at a relatively high rate of 2.9% to 4% [34]. In this study, no cases of deep infection or prosthesis failure were observed in both the controlled and the uncontrolled groups.

The modification of anti-rheumatic medications in the perioperative period has important implications for wound healing. The biological mechanisms underlying this interaction have been reported to be multifactorial. First, DMARDs and biologic agents attenuate the early inflammatory phase of wound healing by suppressing neutrophil and macrophage activity, which may delay the bacterial clearance and debridement of necrotic tissue [35]. In addition, the inhibition of pro-inflammatory cytokines, such as tumor necrosis factor-α (TNF-α), Interleukin-1 (IL-1), and IL-6, reduces the downstream release of growth factors, including transforming growth factors-β (TGF-β) and vascular endothelial growth factor (VEGF), thereby impairing fibroblast proliferation, collagen deposition, and angiogenesis [36,37]. Antimetabolite DMARDs, such as methotrexate and leflunomide, further compromise cellular proliferation and extracellular matrix synthesis, leading to delayed tissue regeneration [36]. Moreover, the immunosuppressive effects of these drugs can diminish immune surveillance, predisposing patients to postoperative infection and secondary impairment of wound closure [36]. Concomitant corticosteroid therapy may exacerbate these risks by inhibiting fibroblast activity and collagen synthesis, as well as inducing metabolic disturbances such as hyperglycemia [38]. In summary, these biological mechanisms highlight the delicate balance between minimizing disease flare-ups through continued immunosuppression and optimizing wound healing through the temporary modification of anti-rheumatic therapy.

Clinical outcomes after TAA are typically assessed using the American Orthopaedic Foot and Ankle Society (AOFAS) score, with previous mid-term studies reporting average scores between 73 and 86 [39,40]. In this study, the average AOFAS score at the 2-year follow-up was 82.3 for the controlled group and 81.8 for the uncontrolled group, consistent with the previous findings. However, the AOFAS Ankle–Hindfoot score has limitations due to its relatively simplistic structure in evaluating functional and ambulatory capacity. To address these shortcomings, this study also included a patient-reported outcome measure (FAOS) which consists of 42 questions. This tool provided more detailed assessments of the patients’ functional status in daily living and sports participation. At the 2-year follow-up, the FAOS subscore for daily living activities averaged 84.9 for the controlled group and 82.8 for the uncontrolled group, while the sports subscore was 58.2 and 55.6, respectively. Although both clinical evaluation scores (AOFAS and FAOS) showed significant improvement compared to preoperative values, participation in sport activities remained limited relative to improvements in daily function.

Regarding prosthesis survival, Fevang et al. [41] reported 5-year and 10-year survival rates of 89% and 76%, respectively, in RA patients undergoing TAA. While San Giovanni et al. [42] reported a survival rate of 93% at a mean of 8.3 years. It has been noted that RA patients exhibit higher rates of aseptic loosening and polyethylene insert dislocation than those with degenerative or post-traumatic arthritis [12]. In this study, aside from two cases of implant subsidence, no abnormal findings, such as aseptic loosening or polyethylene insert subluxation, were observed.

Persistent pain following TAA has been reported in a substantial proportion of patients [43], often due to malpositioned implants causing unbalanced loading across the joint. In this study, two patients exhibited an implant malalignment exceeding 10 degrees. However, they reported significant pain relief during gait, with a visual analogue scale (VAS) score of 2 or less, while three patients experienced persistent medial ankle pain due to soft tissue or spur impingement, which required arthroscopic gutter debridement and spur trimming at 8–14 months postoperatively.

The clinical and radiological results may vary depending on the kind of TAA implant system used. Depending on the TAA systems, there is a variety of range of component constraint, extent of bony resection, and necessity of cement or screw fixation. These distinguishing characteristics between TAA designs can result in differences in osseous integration, osteolysis, implant loosening, polyethylene (PE) insert dislocation, and poor long-term survivorship [3,32]. Early first- and second-generation TAA systems were associated with high rates of loosening and failure, which limited their clinical use. By contrast, third-generation systems have demonstrated improved implant survival and better functional outcomes owing to their enhanced fixation methods, enhanced materials, and more anatomical design features [1,8,43]. Two-component designs, although technically less demanding, are known to often show limitations in mobility and higher rates of PE wear compared with three-component systems. Mobile-bearing implants have been reported to demonstrate favorable kinematics and reduced constraint, but concerns remain regarding PE insert dislocation [8,43]. Recent advances continue to focus on improving longevity and restoring physiological joint kinematics. Although contemporary TAA systems demonstrate superior outcomes compared with earlier designs, specific differences among implant systems suggest that implant selection should be tailored according to patient characteristics, deformity severity, and surgeon experience. Further high-quality, comparative studies with long-term follow-up are needed to clarify the influence of TAA implant design on clinical results.

This study has several limitations. First, it was not a randomized prospective controlled trial in patients undergoing TAA for RA. Even if the all surgeries were performed by the same surgeon and were enrolled consecutively, the difference of proficiency in surgical skill or perioperative care according to the assignment of the uncontrolled group in the early study period and the controlled group in the later study period may have affected the results. This can result in potential selection and temporal bias. Second, the follow-up duration of 2 years was short, limiting sufficient evaluation of long-term complications and implant survivorship. Further studies with more high-level evidence are needed to establish standardized perioperative guidelines for anti-rheumatic medications in TAA for end-stage ankle RA. Third, this study included only one kind of TAA implant system and all cases were performed in a single center. These factors limit the applicability of the study findings to other implant designs or surgical settings. The variability among the TAA implant design and details in the surgical procedure may lead to the heterogeneous results. Finally, this study included relatively small cohorts. A definitive conclusion regarding the usefulness of standardized perioperative management of anti-rheumatic medications should be answered through further studies including a large number of patients.

## 5. Conclusions

In patients with end-stage rheumatoid arthritis, third-generation total ankle arthroplasty demonstrated favorable short-term clinical outcomes. Standardized perioperative management of anti-rheumatic medications based on the specified guideline appears to be an effective strategy to reduce early postoperative wound complications associated with RA. Further prospective comparative studies along with long-term follow-up are warranted to more clearly define the clinical utility of the perioperative modification of anti-rheumatic medications in TAA.

## Figures and Tables

**Table 1:** Comparison of the patients’ demographic and clinical characteristics (Mann–Whitney and Fisher exact tests).

	Controlled Group (n = 18)	Uncontrolled Group (n = 16)	*p*-Value
Sex (no. [%])			
Female	16 (88.9)	15 (93.7)	0.615
Male	2 (11.1)	1 (6.3)	
Age at surgery (year) *	62.3 ± 10.1	64.1 ± 9.7	0.784
Body mass index (kg/m^2^) *	25.5 ± 2.8	24.7 ± 2.9	0.695
Bone mineral density (g/cm^2^) *	1.05 ± 0.4	1.03 ± 0.5	0.851
Smoking (no. [%])			
Never	10 (55.6)	8 (50)	0.232
Former	6 (33.3)	7 (43.8)	
Smoker	2 (11.1)	1 (6.3)	
Diabetes (no. [%])	3 (16.7)	2 (12.5)	0.565
Peripheral neuropathy	1 (5.6)	1 (6.3)	0.894
Peripheral vascular disease	1 (5.6)	1 (6.3)	0.894
Hypertension	5 (27.8)	4 (25)	0.902
Osteoporosis (T-score ≤ −2.5)	4 (22.2)	4 (25)	0.631

* Data are given as the mean and standard deviation.

**Table 2:** Comparison of the data regarding the operative procedures (Mann–Whitney and Fisher exact tests).

	Controlled Group (n = 18)	Uncontrolled Group (n = 16)	*p*-Value
Tourniquet time (minutes) *	87.8 ± 22.6	90.1 ± 22.1	0.793
Total ankle replacement only	14 (77.8)	13 (81.3)	0.661
Additional procedures (no. [%])			
Autologous bone graft	3 (16.7)	2 (12.5)	0.565
Lateral ligaments stabilization	2 (11.1)	1 (6.3)	0.427
Gastrocnemius recession	1 (5.6)	0	0.315
Achilles tendon lengthening	1 (5.6)	1 (6.3)	0.894
Calcaneal sliding osteotomy	0	1 (6.3)	0.221
Fixation for malleolar fracture	1 (5.6)	1 (6.3)	0.894

* Data are given as the mean and standard deviation.

**Table 3:** Comparison of the clinical outcomes (Wilcoxon signed-rank test).

	Controlled Group		Uncontrolled Group		*p*-Value ^†^
	Preoperative	PO 2 Years	Preoperative	PO 2 Years	
AOFAS *					
Pain	16.1 ± 6.4	35.6 ± 8.5	15.8 ± 6.6	35.7 ± 8.3	0.981
Function	15.4 ± 7.2	37.2 ± 9.8	15.1 ± 6.9	36.5 ± 9.1	0.709
Alignment	6.1 ± 2.4	9.5 ± 3.7	6.6 ± 2.5	9.6 ± 3.4	0.935
Total scores	37.6 ± 12.5	82.3 ± 16.1	37.5 ± 12.2	81.8 ± 17.4	0.722
FAOS *					
Pain	38.7 ± 10.4	84.8 ± 10.4	39.5 ± 10.6	85.1 ± 10.9	0.887
Other symptoms	44.5 ± 12.2	84.4 ± 10.9	43.1 ± 11.5	83.8 ± 11.8	0.721
Daily living	47.8 ± 14.9	84.9 ± 13.7	48.4 ± 15.5	82.8 ± 13.2	0.314
Sports and leisure	30.9 ± 9.3	58.2 ± 16.2	29.7 ± 9.4	55.6 ± 15.9	0.225
Quality of life	31.5 ± 9.9	85.1 ± 10.1	30.1 ± 9.8	84.5 ± 10.4	0.708
Total scores	38.9 ± 11.3	79.5 ± 17.2	38.2 ± 10.8	78.4 ± 16.1	0.546

Abbreviations: PO, postoperative; AOFAS, American Orthopaedic Foot and Ankle Society; FAOS, Foot and Ankle Outcome Score. * Data are given as the mean and standard deviation. † Comparison of the clinical outcomes at 2 years postoperatively between the two groups.

**Table 4:** Comparison of postoperative complications (Fisher exact tests).

	Controlled Group (n = 18)	Uncontrolled Group (n = 16)	*p*-Value
Marginal skin necrosis (no. [%])	1 (5.6%)	2 (12.5%)	0.116
Delayed wound healing > 4 weeks	0	4 (25%)	**0.0** **39**
Medial malleolar fracture	1 (5.6%)	1 (6.3%)	0.838
Superficial wound infection	0	1 (6.3%)	0.275
Deep wound infection	0	0	N/A
Deep peroneal nerve injury	1 (5.6%)	0	0.403
Malalignment of the implants ≥ 10°			
Tibial component	1 (5.6%)	1 (6.3%)	0.838
Talar component	0	0	N/A
Loosening of the implants			
Tibial (position change > 2°)	0	0	N/A
Talar (position change > 5°)	0	0	N/A
Subsidence into the talus > 5 mm	1 (5.6%)	1 (6.3%)	0.838
Osteolysis (radiolucent line > 2 mm)	0	0	N/A
Subluxation of polyethylene insert	0	0	N/A
Heterotopic ossification	1 (5.6%)	1 (6.3%)	0.838
Soft tissue or spur impingement	2 (11.1%)	1 (6.3%)	0.365
Progressive arthritis in adjacent joint	1 (5.6%)	0	0.403

Statistically significant values are indicated in bold.

**Table 5:** Comparison of postoperative wound healing (Mann–Whitney test).

ASEPSIS Score *	PO 1 Week	PO 2 Weeks	PO 3 Weeks	PO 4 Weeks
Controlled group	19.5 ± 8.8	13.8 ± 6.5	9.2 ± 4.4	3.1 ± 1.6
Uncontrolled group	20.3 ± 9.1	17.1 ± 8.4	14.7 ± 6.9	6.8 ± 3.5
*p*-value	0.891	**0.014**	**<0.001**	**0.003**

Abbreviations: PO, postoperative. * Data are given as the mean and standard deviation. Statistically significant values are indicated in bold.

## Data Availability

The data presented in this study are available in the article.

## Abbreviations

The following abbreviations are used in this manuscript:

RA	Rheumatoid arthritis
TAA	Total ankle arthroplasty
AOFAS	American Orthopaedic Foot and Ankle Society
FAOS	Foot and Ankle Outcome Score
